# Numerical study of the laser isotope separation of optically pumped ^102^Pd

**DOI:** 10.1038/s41598-024-54262-y

**Published:** 2024-02-19

**Authors:** M. V. Suryanarayana, M. Sankari

**Affiliations:** Bhabha Atomic Research Centre, Visakhapatnam, Andhra Pradesh India

**Keywords:** Physics, Atomic and molecular physics, Atomic and molecular interactions with photons

## Abstract

The feasibility of laser isotope separation of ^102^Pd through pulsed laser optical pumping followed by isotope selective photoionization has been studied through density matrix formalism. The effect of various parameters such as bandwidth of the excitation lasers, intensity of the lasers and Doppler broadening of the atomic ensemble on the efficiency of optical pumping and isotope selective photoionization have been evaluated. The optimum number density in the laser-atom interaction region has been derived from the studies of the effect of charge exchange collisions on the degree of enrichment. It has been shown that it is possible to enrich ^102^Pd up to ~ 23.7% at a production rate of 1.1 mg /h. The achievable degree of enrichment through this photoionization scheme is higher than the previously reported laser isotope separation process. The radionuclidic purity of the irradiated enriched mixture has been found to be suitable for medical applications.

## Introduction

Palladium has six stable isotopes (Table 1), namely, ^102^Pd (natural abundance = 1.02%), ^104^Pd (natural abundance = 11.1%), ^105^Pd (natural abundance = 22.3%), ^106^Pd (natural abundance = 27.3%), ^108^Pd (natural abundance = 26.5%) and ^110^Pd (natural abundance = 11.7%). Currently, palladium metal costs about 40,000 USD/kg. Pd is also produced in the nuclear reactor as a fission product. Due to its high value, separation of Pd from the nuclear reactor spent fuel has been studied^1,2^. Apart from the difficulties in the chemical separation of Pd from the spent fuel, the primary limitation lies due to the presence of the long-lived ^107^Pd (T_1/2_ = 6.5 × 10^6^ years) isotope in the nuclear reactor spent fuel. Laser isotope separation method has been studied^3^ for the removal ^107^Pd from the palladium recovered from nuclear reactor spent fuel. At present, cost of laser cleanup of reactor produced Pd limits practical utility of the method.

**Table 1:** Table of atomic and nuclear parameters of Pd isotopes.

Isotope	Natural abundance (%)	Nuclear spin	Change in mean square nuclear charge radii (fm^2^) relative to ^102^Pd^4^	Half-life (T_1/2_)^5^	Magnetic moment μ (nm)^6^	Quadrupole moment Q (b)^6^	Thermal neutron absorption cross-section (barns)^5^
^102^Pd	1.02	–	0	–	–	–	1.82
^103^Pd	–	5/2	–	16.991 d	NA	NA	NA
^104^Pd	11.1	–	0.2318	–	–	–	0.65
^105^Pd	22.3	5/2	0.3013	–	–0.642	+ 0.660	21
^106^Pd	27.3	–	0.4505	–	–	–	0.305
^107^Pd	–	5/2	–	6.5 × 10^6^ y	NA	NA	9.5
^108^Pd	26.5	–	0.6782	–	–	–	8.57
^109^Pd	–	5/2	NA	13.59 h	NA	NA	NA
^110^Pd	11.7	–	0.8834	–	–	–	0.30

*NA* not available.

The radioisotope ^103^Pd (T_1/2_ = 16.991 days) decays 100% by electron capture to ^103m^Rh by emitting two characteristic X-rays with energy 20.073 (22.09%) keV and 20.215 (41.83%) keV. The Q-value of the reaction is + 543.1 keV. Due to its favorable decay properties, ^103^Pd is used for radiation therapy of the patients suffering from prostate cancer^7^ and uveal melanoma^8^. ^103^Pd is produced by irradiation of its precursor ^102^Pd isotope in a nuclear reactor. Owing to the low natural abundance of the parent ^102^Pd isotope and low thermal neutron absorption cross-section^9^ (σ_th_ = 1.82 b), the amount of ^103^Pd isotope produced in the nuclear reactor would be rather small. As a result, the specific activity of the produced ^103^Pd isotope would also be small. For example, when one gram of natural Pd is irradiated in a low-flux nuclear reactor (neutron flux = 3 × 10^13^ neutrons/cm^2^-sec) for 60 days, at the end of irradiation, the amount of ^103^Pd produced is ~ 1 μg. Though the no-carrier added specific activity of ^103^Pd (2.76 × 10^15^ Bq/gram) is high, the specific activity of the ^103^Pd produced from the natural Pd (2.76 × 10^9^ Bq/gram) is rather low. Further, during the irradiation, the highly abundant ^106^Pd (27.3%) and ^108^Pd (26.5%) isotopes produce ^107^Pd (T_1/2_ = 6.5 × 10^6^ years) and ^109^Pd (T_1/2_ = 13.59 h) radioactive daughter isotopes respectively which degrades the radionuclidic purity of ^103^Pd. The radionuclidic purity of an isotopic mixture is defined by the equation

1 $${R}_{i} (\%)= \frac{{S}_{i}*{f}_{i}}{\sum_{i}^{n}{S}_{i}*{f}_{i}}\times 100$$

where *S*_*i*_ = Specific activity of the radioisotope “i” (Bq/gm), *f*_*i*_ = Relative fractional abundance of the radioisotope in the isotopic mixture, n = Number of radioisotopes in the isotopic mixture.

No-carrier added specific activity of a radioisotope can be calculated using the following expression

2 $$S= \frac{4.174\times {10}^{23}}{{AMU\times T}_{1/2}}$$

where S = Specific activity (Bq/gm), AMU = Atomic weight of the isotope (amu) and T_1/2_ = Half-life of the isotope (s).

At the end of irradiation, the radionuclidic purity of ^103^Pd in the irradiated isotopic mixture is 0.78% while the radionuclidic purity of ^109^Pd is 99.21%. Since both isotopes have widely varying half-lives (Table 1), patient dose optimization is a complex task. This necessitates the utilization of enriched ^102^Pd for the production of ^103^Pd medical isotope.

Laser enrichment of ^102^Pd isotope is extremely complex owing to its low natural abundance, high melting (1554.9 °C) and boiling (2963 °C) points and large ionization potential (67,241.14 cm^−1^ or 8.3368 eV). Additionally, all the three known transitions viz., ^1^S_0_–^1^P°_1_ (244.8647 nm), ^1^S_0_–^3^D°_1_ (247.7161 nm) and ^1^S_0_–^3^P°_1_ (276.3906 nm) originating from the 4d^10 1^S_0_ (0.0 cm^−1^) ground state have their wavelengths in the UV region and their isotopes shifts have been reported to be extremely small^10^. In general, for the mid-Z elements, the field shift (also known as volume shift) and the mass shift are of comparable magnitudes. When these two components are in opposing direction, the net isotope shift, which is a sum of field shift and the mass shift, would be rather small (Table 2). This makes laser isotope separation of ^102^Pd a daunting task.

**Table 2:** Isotope shifts of the transitions of Pd isotopes.

Transition	Isotope shift with reference to ^102^Pd (MHz)						References
	^104^Pd	^105^Pd	^106^Pd	^107^Pd	^108^Pd	^110^Pd	
276.3906 nm	− 69.4	−	− 142.9	−	− 205.6	− 272	^10^
361.0575 nm	− 465.2	− 568.9	− 927.4	−	− 1413.6	− 1843	^11^

Researchers at the AM Prokhorov General Physics Institute^12–14^, Russia have employed the following photoionization scheme for the laser isotope separation of ^102^Pd.

$$4{d}^{10}{}^{1}{S}_{0} \left(0.0\;{{\text{cm}}}^{-1}\right) \stackrel{276.3906\;{\text{nm}}}{\to }4{d}^{9}5p {}^{3}{P}_{1}^{o}\left(36180.677\;{{\text{cm}}}^{-1}\right) \stackrel{291\;{\text{nm}}}{\to }\;{{\text{Pd}} }^{+}$$

They have reported that the bandwidth of the excitation lasers needs to be controlled to about 60–80 MHz for obtaining 18% enrichment of ^102^Pd isotope^12^. Further, the angular divergence of the atomic beam shall be restricted to 0.1 rad (5.7°) for the requisite isotopic selectivity. Additionally, to minimize the overlap of the hyperfine spectrum of the^105^Pd isotope, a magnetic field of 2000G has been employed in the laser-atom interaction region. On the whole, the experimental setup is complex and hence the reported method^12^ may not be suitable for laser based separations where the volume of laser-atom interaction region is large.

Sarina Geldhof et al.^15^ have recently measured isotope shifts of the natural isotopes and the hyperfine structure of the odd ^105^Pd isotope for the transitions originating from the 4d^10 1^S_0_ (0.0 cm^−1^) ground state and the 4d^9^5s ^3^D_3_ (6564.148 cm^−1^), 4d^9^5s ^3^D_2_ (7755.025 cm^−1^), 4d^9^5s ^3^D_1_ (10,093.992 cm^−1^) and 4d^9^5s ^1^D_2_ (11,721.809 cm^−1^) meta-stable states. The isotope shifts of the transitions measured were found to be much higher than the 276.3906 nm previously used for laser isotope separation of Pd (Table 2).

**Figure 1:**
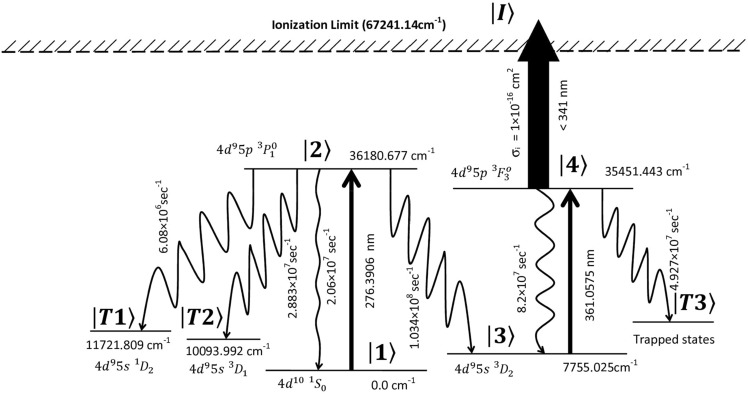
Schematic diagram of the photoionization scheme of palladium (not to scale).

Upon careful observation of the branching ratios and the decay rates of the transitions (Fig. 1), it can be observed that the 276.3906 nm transition can be used for the efficient optical pumping of atoms from the 4d^10 1^S_0_ (0.0 cm^−1^) ground state into the 4d^9^5s ^3^D_2_ (7755.025 cm^−1^) meta-stable state. Due to the large isotope shift, these optically pumped atoms can be selectively photoionized using the 361.0575 nm transition.

The photoionization process is shown below.

### Optical pumping

$$4{d}^{10}{}^{1}{S}_{0} \left(0.0\;{{\text{cm}}}^{-1}\right) \stackrel{276.3906\;{\text{nm}}}{\to }\;4{d}^{9}5p {}^{3}{P}_{1}^{o}\left(36180.677\;{{\text{cm}}}^{-1}\right)\;\stackrel{radiative\;decay}\;{\to }\;4{d}^{9}5s {}^{3}{D}_{2} \left(7755.025\;{{\text{cm}}}^{-1}\right)$$

### Photoionization

$$\begin{array}{c}4{d}^{9}5s {}^{3}{D}_{2} \left(7755.025\;{{\text{cm}}}^{-1}\right)\;\stackrel{361.0575\;{\text{nm}}}{\to }\;4{d}^{9}5p {}^{3}{F}_{3}^{o} \left(35451.443\;{{\text{cm}}}^{-1}\right) \\ \stackrel{< 341\;{\text{nm}}}\;{\to }\;{Pd}^{+}\end{array}$$

**Figure 2:**
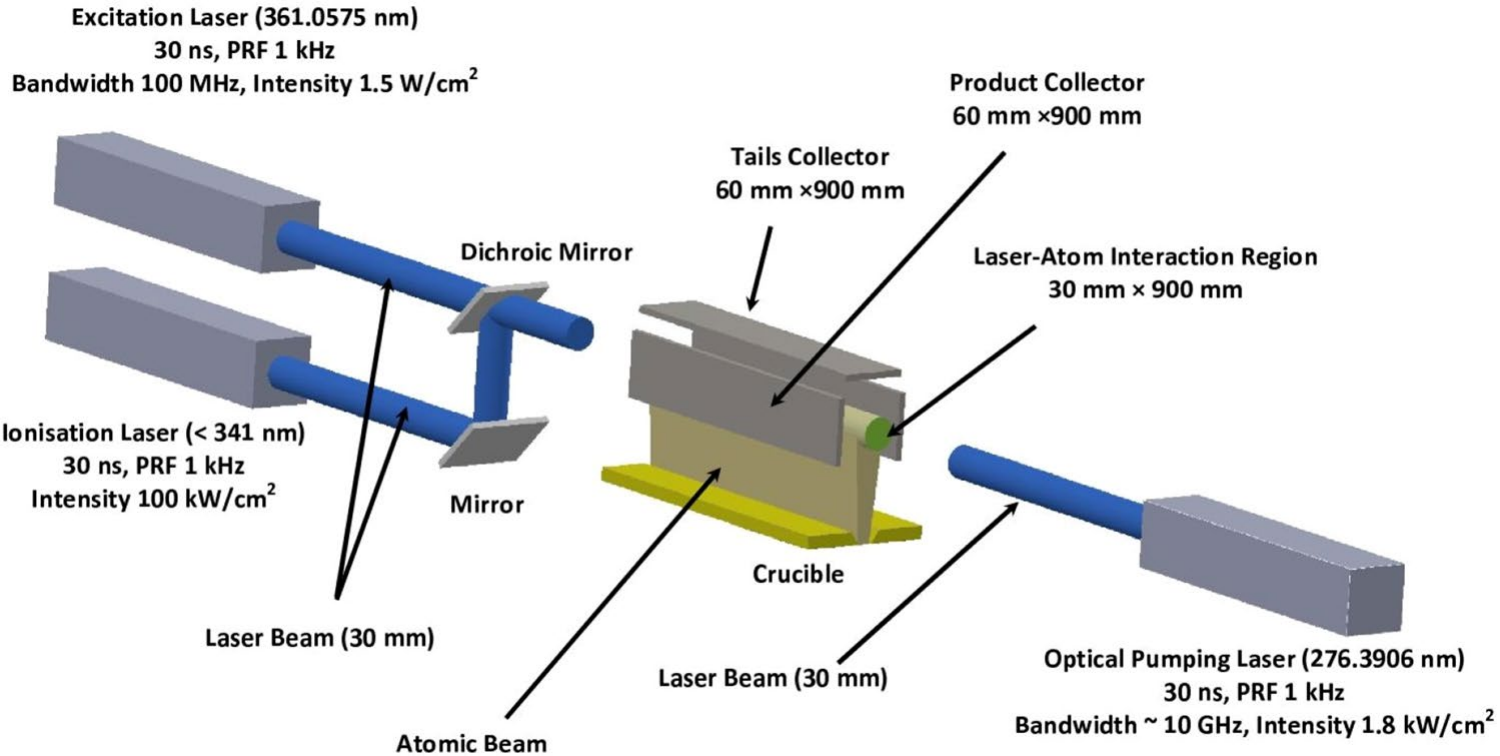
Schematic of the experimental geometry for the laser isotope separation of Pd.

In the present work, the photoionization process mentioned above has been studied for its suitability for the laser isotope separation of ^102^Pd. A schematic of the proposed experimental setup is shown in Fig. 2. The density matrix formalism accurately describes the laser-atom interactions^16^ in the multi-step laser excitation processes; therefore, it has been invoked for the calculation of degree of enrichment and production rates of the laser isotope separation process under various conditions. The optimization of the laser isotope separation process has been done numerically.

### Theoretical basis

Palladium has a ground state configuration of 4d^10 1^S_0_ (0.0 cm^−1^). At 1500 °C, 96% of the population is available in the ground state. At a temperature (T^0^ K), the vapour pressure can be calculated using the following equation^17^,

3 $$\mathrm{log }(P)= A+ \left(\frac{B}{T}\right)+C{\text{log}}(T)+ \left(\frac{D}{{T}^{3}}\right)$$

where P = Vapor pressure (bar). For the case of elemental Pd^13^, A = 5.426, B = − 17,889, C = 0 and D = 0.

At 1500 °C, the vapor pressure of Pd is calculated to be 22 μbar which corresponds to a number density of 9 × 10^13^ atoms/cm^3^.

The population of the ground state can be excited into the 4d^9^5p ^3^P°_1_ state using a nanosecond pulsed broadband laser tuned to the 276.3906 nm transition (Fig. 1).

$$4{d}^{10}{}^{1}{S}_{0} \left(0.0\;{{\text{cm}}}^{-1}\right)\;\stackrel{276.3906\;{\text{nm}}}\;{\to }\;4{d}^{9}5p {}^{3}{P}_{1}^{o}\left(36180.677\;{{\text{cm}}}^{-1}\right)\;\stackrel{radiative\;decay}{\to }\;4{d}^{9}5s {}^{3}{D}_{2} \left(7755.025\;{{\text{cm}}}^{-1}\right)$$

Due to the finite lifetime^18^ of the upper level 4d^9^5p ^3^P°_1_ (τ_1/2_ = 7.6 ns), the atoms decay to the 4d^10 1^S_0_ (0.0 cm^−1^) ground state and the 4d^9^5s ^3^D_2_ (7755.025 cm^−1^), 4d^9^5s ^3^D_1_ (10,093.992 cm^−1^), 4d^9^5s ^1^D_2_ (11,721.809 cm^−1^) high-lying meta-stable states. Due to the high branching fraction (0.65), most of the population decays to the 4d^9^5s ^3^D_2_ (7755.025 cm^−1^).

The atom dynamics of the optical pumping process can be described by the coupled density matrix equations described below. The atoms initially present in the ground fine-structure level $\left. {\left| 1 \right.} \right\rangle$ are optical pumped into the level $\left. {\left| 2 \right.} \right\rangle$. Due to the finite life-time, the atoms in level $\left. {\left| 2 \right.} \right\rangle$ decay to the high-lying meta-stable level $\left. {\left| 3 \right.} \right\rangle$ and to the other trapping levels $\left. {\left| {T1} \right.} \right\rangle$ and $\left. {\left| {T2} \right.} \right\rangle$. The atoms in the resonant level $\left. {\left| 2 \right.} \right\rangle$ may also decay to the resonant lower level $\left. {\left| 1 \right.} \right\rangle$ at a rate denoted as Γ_21_. The decay rate of atoms from the resonant level $\left. {\left| 2 \right.} \right\rangle$ to trapped levels $\left. {\left| {T1} \right.} \right\rangle$ and $\left. {\left| {T2} \right.} \right\rangle$ at a total rate denoted as γ_2T_.

4 $$\dot{\uprho }\left(1,i,1,i\right)=-i.\left\{\sum_{j=1}^{J}{\Omega }_{1}^{*}\left(1,i,2,j\right).\uprho \left(1,{\text{i}},2,{\text{j}}\right)\right\}+i.\left\{\sum_{j=1}^{J}{\Omega }_{1}\left(1,i,2,j\right).\uprho \left(2,{\text{j}},1,{\text{i}}\right)\right\} +2.\left\{\sum_{j=1}^{J}{\Gamma }_{21}\left(1,i,2,j\right).\uprho \left(2,{\text{j}},2,{\text{j}}\right)\right\}$$

5 $$\dot{\uprho }\left(2,j,2,j\right)=-i.\left\{\sum_{i=1}^{I}{\Omega }_{1}\left(1,i,2,j\right).\uprho \left(2,{\text{j}},1,{\text{i}}\right)\right\}+i.\left\{\sum_{i=1}^{I}{\Omega }_{1}^{*}\left(1,i,2,j\right).\uprho \left(1,{\text{i}},2,{\text{j}}\right)\right\}-2.\left\{\sum_{i=1}^{I}{\Gamma }_{21}\left(1,i,2,j\right).\uprho \left(2,{\text{j}},2,{\text{j}}\right)\right\}-2.\left\{({\gamma }_{23}+{\gamma }_{2T}).\uprho \left(2,{\text{j}},2,{\text{j}}\right)\right\}$$

6 $$\begin{gathered} \dot{\uprho }\left( {1,i,2,j} \right) = - i.\left\{ {\mathop \sum \limits_{i1 = 1}^{I} \Omega_{1} \left( {1,i1,2,j} \right).\uprho \left( {1,i1,1,i1} \right)} \right\} + i.\left\{ {\mathop \sum \limits_{j1 = 1}^{J} \Omega_{1} \left( {1,i,2,j1} \right).\uprho \left( {2,j1,2,j1} \right)} \right\} \hfill \\ \quad \quad \quad \quad \quad \quad - \left\{ {\left[ {i.\Delta \left( {1,i,2,j} \right) + \gamma_{L} \left( {1,2} \right) + \gamma_{23} + \gamma_{2T} + \Gamma_{21} \left( {1,i,2,j} \right)} \right].\uprho \left( {1,i,2,j} \right)} \right\} \hfill \\ \end{gathered}$$

7 $$\begin{gathered} {\dot{\uprho }}\left( {2,j,1,i} \right) = { }i.\left\{ {\mathop \sum \limits_{i1 = 1}^{I} {\Omega }_{1}^{*} \left( {1,i1,2,j} \right).{\uprho }\left( {1,{\text{i}}1,1,{\text{i}}1} \right)} \right\}{ } - i.\left\{ {\mathop \sum \limits_{j1 = 1}^{J} {\Omega }_{1}^{*} \left( {1,i,2,j1} \right).{\uprho }\left( {2,{\text{j}}1,2,{\text{j}}1} \right)} \right\} \hfill \\ \quad \quad \quad \quad \quad \quad - { }\left\{ {\left[ { - {\text{i}}.\Delta \left( {1,i,2,j} \right) + \gamma_{L} \left( {1,2} \right) + \gamma_{23} + \gamma_{2T} + \Gamma_{21} \left( {1,i,2,j} \right)} \right].{\uprho }\left( {2,j,1,i} \right)} \right\} \hfill \\ \end{gathered}$$

8 $$\dot{\uprho }\left(1,i,1,i1\right)=-i.\left\{\sum_{j=1}^{J}{\Omega }_{1}^{*}\left(1,i\mathrm{1,2},j\right).\uprho \left(1,{\text{i}},2,{\text{j}}\right)\right\}+i\left\{\sum_{j=1}^{J}{\Omega }_{1}\left(1,i,2,j\right).\uprho \left(2,{\text{j}},1,{\text{i}}1\right)\right\}-\left\{\sum_{j=1}^{J}{\Gamma }_{21}\left(1,i,2,j\right).\uprho \left(1,{\text{i}},1,{\text{i}}1\right)\right\}-\left\{\sum_{j=1}^{J}{\Gamma }_{21}\left(1,i\mathrm{1,2},j\right).\uprho \left(1,{\text{i}},1,{\text{i}}1\right)\right\}$$

9 $$\dot{\uprho }\left(1,i\mathrm{1,1},i\right)=i.\left\{\sum_{j=1}^{J}{\Omega }_{1}\left(1,i\mathrm{1,2},j\right).\uprho \left(2,{\text{j}},1,{\text{i}}\right)\right\}-i.\left\{\sum_{j=1}^{J}{\Omega }_{1}^{*}\left(1,i,2,j\right).\uprho \left(1,\mathrm{i1,2},{\text{j}}\right)\right\}-\left\{\sum_{j=1}^{J}{\Gamma }_{21}\left(1,i,2,j\right).\uprho \left(1,i\mathrm{1,1},i\right)\right\}-\left\{\sum_{j=1}^{J}{\Gamma }_{21}\left(1,i\mathrm{1,2},j\right).\uprho \left(1,i\mathrm{1,1},i\right)\right\}$$

10 $$\dot{\uprho }\left(2,j,2,j1\right)=-i.\left\{\sum_{i=1}^{I}{\Omega }_{1}\left(1,i,2,j1\right).\uprho \left(2,{\text{j}},1,{\text{i}}\right)\right\}+ i.\left\{\sum_{i=1}^{I}{\Omega }_{1}^{*}\left(1,i,2,j\right).\uprho \left(1,{\text{i}},2,{\text{j}}1\right)\right\}-\left\{\sum_{i=1}^{I}{\Gamma }_{21}\left(1,i,2,j\right).\uprho \left(2,j,2,j1\right)\right\}-\left\{\sum_{i=1}^{I}{\Gamma }_{21}\left(1,i,2,j1\right).\uprho \left(2,j,2,j1\right)\right\}- \left\{\left[{\gamma }_{23}+{\gamma }_{2T}\right].\uprho \left(2,j,2,j1\right)\right\}$$

11 $$\dot{\uprho }\left(2,j\mathrm{1,2},j\right)=i\left\{\sum_{i=1}^{I}{\Omega }_{1}^{*}\left(1,i,2,j1\right).\uprho \left(1,{\text{i}},2,{\text{j}}\right)\right\}- i.\left\{\sum_{i=1}^{I}{\Omega }_{1}\left(1,i,2,j\right).\uprho \left(2,\mathrm{j1,1},{\text{i}}\right)\right\}-\left\{\sum_{i=1}^{I}{\Gamma }_{21}\left(1,i,2,j\right).\uprho \left(2,j\mathrm{1,2},j\right)\right\}-\left\{\sum_{i=1}^{I}{\Gamma }_{21}\left(1,i,2,j1\right).\uprho \left(2,j\mathrm{1,2},j\right)\right\}- \left\{\left[{\gamma }_{23}+{\gamma }_{2T}\right].\uprho \left(2,j\mathrm{1,2},j\right)\right\}$$

12 $$\dot{\uprho }\left(\mathrm{3,1},\mathrm{3,1}\right)=\left\{2.\sum_{j=1}^{J}{\gamma }_{23}.\uprho \left(2,{\text{j}},2,{\text{j}}\right)\right\}$$

13 $$\dot{\uprho }\left(T,1,T,1\right)=\left\{2.\sum_{j=1}^{J}{\gamma }_{2T}.\uprho \left(2,{\text{j}},2,{\text{j}}\right)\right\}$$

where the density matrix element $\uprho \left(M,{\text{m}},{\text{N}},{\text{n}}\right)$ describes the coherence between the states $\left. {\left| {M,m} \right.} \right\rangle$ and $\left. {\left| {N,n} \right.} \right\rangle$ when M ≠ N and/or m ≠ n and represents the level population when M = N and m = n. Δ corresponds to the detuning of the laser frequency from the resonance.

The laser is considered to be having a pulse width of 30 ns and a pulse repetition frequency (PRF) of 1 kHz. To include laser bandwidth effects in the calculations the optical pumping laser is considered to have a phase diffusion bandwidth. The laser bandwidth and its line shape have been included in the terms

14 $${\gamma }_{L}={\gamma }_{L}\frac{{\beta }_{1}^{2}}{{\Delta }_{1}^{2}+{\beta }_{1}^{2}}$$

From the above equation, the bandwidth of the lasers at the atomic resonance corresponds to γ_L_; and at large detunings, the laser is nearly monochromatic.

Since the optical pumping laser has a temporal intensity profile, the time varying Rabi frequency has been calculated according to the equation

15 $$\Omega \left(t\right)=\Omega .\sqrt{I(t)}$$

where I(t) is the temporal profile of the laser intensity; Ω is the time-independent Rabi frequency.

As discussed earlier, the population of the meta-stable level 4d^9^5s ^3^D_2_ (7755.025 cm^−1^) can be photoionized through the pathway shown below.

$$\begin{array}{c}4{d}^{9}5s {}^{3}{D}_{2} \left(7755.025 {{\text{cm}}}^{-1}\right) \stackrel{361.0575 {\text{nm}}}{\to }4{d}^{9}5p {}^{3}{F}_{3}^{o} \left(35451.443 {{\text{cm}}}^{-1}\right) \\ \stackrel{< 341 {\text{nm}}}{\to } {Pd}^{+}\end{array}$$

The atoms optically pumped into the level $\left. {\left| 3 \right.} \right\rangle$ are excited by the excitation laser to the level $\left. {\left| 4 \right.} \right\rangle$. The atoms from this level are incoherently excited into the ionization level $\left. {\left| I \right.} \right\rangle$ by the ionization laser at a rate denoted as γ_I_. The atoms in level $\left. {\left| 4 \right.} \right\rangle$ may decay to the lower level $\left. {\left| 3 \right.} \right\rangle$ at a rate denoted as Γ_43_. The atoms in the level $\left. {\left| 4 \right.} \right\rangle$ also decay to the trapped level $\left. {\left| {T3} \right.} \right\rangle$ at a rate denoted as γ_4T_ and are lost from the excitation process. The population dynamics corresponding to the photoionization process of an odd isotope can be described by the coupled density matrix equations given below.

16 $$\dot{\uprho }\left(3,i,3,i\right)=-i.\left\{\sum_{j=1}^{J}{\Omega }_{2}^{*}\left(3,i,4,j\right).\uprho \left(3,{\text{i}},4,{\text{j}}\right)\right\}+i.\left\{\sum_{j=1}^{J}{\Omega }_{2}\left(3,i,4,j\right).\uprho \left(4,{\text{j}},3,{\text{i}}\right)\right\} +2.\left\{\sum_{j=1}^{J}{\Gamma }_{43}\left(3,i,4,j\right).\uprho \left(4,{\text{j}},4,{\text{j}}\right)\right\}$$

17 $$\dot{\uprho }\left(4,j,4,j\right)=-i.\left\{\sum_{i=1}^{I}{\Omega }_{2}\left(3,i,4,j\right).\uprho \left(4,{\text{j}},3,{\text{i}}\right)\right\}+i.\left\{\sum_{i=1}^{I}{\Omega }_{2}^{*}\left(3,i,4,j\right).\uprho \left(3,{\text{i}},4,{\text{j}}\right)\right\}-2.\left\{\sum_{i=1}^{I}{\Gamma }_{43}\left(3,i,4,j\right).\uprho \left(4,{\text{j}},4,{\text{j}}\right)\right\}-2.\left\{\left[{{\gamma }_{4T}+\gamma }_{I}\right].\uprho \left(4,{\text{j}},4,{\text{j}}\right)\right\}$$

18 $$\dot{\uprho }\left(3,i,4,j\right)=-i.\left\{\sum_{i1=1}^{I}{\Omega }_{2}\left(3,i\mathrm{1,4},j\right).\uprho \left(3,\mathrm{i1,3},{\text{i}}1\right)\right\}+i.\left\{\sum_{j1=1}^{J}{\Omega }_{2}\left(3,i,4,j1\right).\uprho \left(4,\mathrm{j1,4},{\text{j}}1\right)\right\}- \left\{\left[{\text{i}}.\Delta \left(3,i,4,j\right)+{\gamma }_{L}\left(\mathrm{3,4}\right)+{\gamma }_{4T}+{\gamma }_{I}+{\Gamma }_{43}\left(3,i,4,j\right)\right].\uprho \left(3,i,4,j\right)\right\}$$

19 $$\dot{\uprho }\left(4,j,3,i\right)= i.\left\{\sum_{i1=1}^{I}{\Omega }_{2}^{*}\left(3,i\mathrm{1,4},j\right).\uprho \left(3,\mathrm{i1,3},{\text{i}}1\right)\right\} -i.\left\{\sum_{j1=1}^{J}{\Omega }_{2}^{*}\left(3,i,4,j1\right).\uprho \left(4,\mathrm{j1,4},{\text{j}}1\right)\right\} - \left\{\left[-{\text{i}}.\Delta \left(3,i,4,j\right)+{\gamma }_{L}\left(\mathrm{3,4}\right)+{\gamma }_{4T}+{\gamma }_{I}+{\Gamma }_{43}\left(3,i,4,j\right)\right].\uprho \left(4,j,3,i\right)\right\}$$

20 $$\dot{\uprho }\left(3,i,3,i1\right)=-i.\left\{\sum_{j=1}^{J}{\Omega }_{2}^{*}\left(3,i\mathrm{1,4},j\right).\uprho \left(3,{\text{i}},4,{\text{j}}\right)\right\}+i\left\{\sum_{j=1}^{J}{\Omega }_{2}\left(3,i,4,j\right).\uprho \left(4,{\text{j}},3,{\text{i}}1\right)\right\}-\left\{\sum_{j=1}^{J}{\Gamma }_{43}\left(3,i,4,j\right).\uprho \left(3,{\text{i}},3,{\text{i}}1\right)\right\}-\left\{\sum_{j=1}^{J}{\Gamma }_{43}\left(3,i\mathrm{1,4},j\right).\uprho \left(3,{\text{i}},3,{\text{i}}1\right)\right\}$$

21 $$\dot{\uprho }\left(3,i\mathrm{1,3},i\right)=i.\left\{\sum_{j=1}^{J}{\Omega }_{2}\left(3,i\mathrm{1,4},j\right).\uprho \left(4,{\text{j}},3,{\text{i}}\right)\right\}-i.\left\{\sum_{j=1}^{J}{\Omega }_{2}^{*}\left(3,i,4,j\right).\uprho \left(3,\mathrm{i1,4},{\text{j}}\right)\right\}-\left\{\sum_{j=1}^{J}{\Gamma }_{43}\left(3,i,4,j\right).\uprho \left(3,i\mathrm{1,3},i\right)\right\}-\left\{\sum_{j=1}^{J}{\Gamma }_{43}\left(3,i\mathrm{1,4},j\right).\uprho \left(3,i\mathrm{1,3},i\right)\right\}$$

22 $$\dot{\uprho }\left(4,j,4,j1\right)=-i.\left\{\sum_{i=1}^{I}{\Omega }_{2}\left(3,i,4,j1\right).\uprho \left(4,{\text{j}},3,{\text{i}}\right)\right\}+ i.\left\{\sum_{i=1}^{I}{\Omega }_{2}^{*}\left(3,i,4,j\right).\uprho \left(3,{\text{i}},4,{\text{j}}1\right)\right\}-\left\{\sum_{i=1}^{I}{\Gamma }_{43}\left(3,i,4,j\right).\uprho \left(4,j,4,j1\right)\right\}-\left\{\sum_{i=1}^{I}{\Gamma }_{43}\left(3,i,4,j1\right).\uprho \left(4,j,4,j1\right)\right\}- \left\{\left[{\gamma }_{4T}+{\gamma }_{I}\right].\uprho \left(2,j,2,j1\right)\right\}$$

23 $$\dot{\uprho }\left(4,j\mathrm{1,4},j\right)=i\left\{\sum_{i=1}^{I}{\Omega }_{2}^{*}\left(3,i,4,j1\right).\uprho \left(3,{\text{i}},4,{\text{j}}\right)\right\}- i.\left\{\sum_{i=1}^{I}{\Omega }_{2}\left(3,i,4,j\right).\uprho \left(4,\mathrm{j1,3},{\text{i}}\right)\right\}-\left\{\sum_{i=1}^{I}{\Gamma }_{43}\left(3,i,4,j\right).\uprho \left(4,j\mathrm{1,4},j\right)\right\}-\left\{\sum_{i=1}^{I}{\Gamma }_{43}\left(3,i,4,j1\right).\uprho \left(4,j\mathrm{1,4},j\right)\right\}- \left\{\left[{\gamma }_{4T}+{\gamma }_{I}\right].\uprho \left(4,j\mathrm{1,4},j\right)\right\}$$

24 $$\dot{\uprho }\left(T\mathrm{3,1},T\mathrm{3,1}\right)=\left\{2.\sum_{i=1}^{I}{\gamma }_{4T3}.\uprho \left(4,{\text{j}},4,{\text{j}}\right)\right\}$$

25 $$\dot{\uprho }\left(I,1,I,1\right)=\left\{2.\sum_{j=1}^{J}{\gamma }_{I} .\uprho \left(4,{\text{j}},4,{\text{j}}\right)\right\}$$

The ionization rate which is induced by the ionizing laser can be calculated using the equation ${\gamma }_{I}= \sigma \phi$, where σ = Photoionization cross-section and ϕ is the flux of the ionization laser. The photoionization cross-section is considered to be 1 × 10^–16^ cm^2^ and the corresponding ionization rate has been calculated to be 0.128 × I kHz (where, I = Intensity of the laser in W/cm^2^).

The coupled differential equations are integrated using standard numerical integration methods.

## Doppler broadening and atomic flux-velocity distribution

The most probable velocity (v_mp_) of the atoms can be calculated using the expression

26 $${v}_{mp}= \sqrt{\frac{2kT}{m}}$$

where *k* is the Boltzmann Constant (1.380649 × 10^–23^ J/K), *T* is the temperature of the atomic ensemble (°K) and *m* is the mass of the atom (kg). At 1500 °C, the most probable atomic velocity of ^102^Pd isotope is 537.507 m/s. The variation in the mean probable velocity between the stable Pd isotopes is ± 8 m/s. At this temperature, a variation of ± 50 °C in the temperature results in ± 8 m/s variation in the most probable velocity. These small variations in the velocities do not induce any significant effect on the laser isotope separation process.

Doppler broadening of the atomic transitions arises due to the velocity and angular distributions of atoms effusing from the atom source. The flux-velocity distribution of atomic ensemble can be described by the following expression

27 $$\phi \left(v\right)=2\left(\frac{{v}^{3}}{{v}_{mp}^{4}}\right).{e}^{-\left(\frac{{v}^{2}}{{v}_{mp}^{2}}\right)}dv$$

where, v_mp_ = Most probable velocity. At 4 × v_mp_, the relative flux drops to the value of ~ 10^–7^ of the maximum.

The Doppler broadening of an atomic transition can be calculated using the expression

28 $$\Delta {\nu }_{D}(Hz)=7.162\times {10}^{-7}.{\nu }_{0}.\sqrt{\frac{T}{M}}$$

where ν_0_ is the resonance frequency of the transition (Hz), T is the temperature of the atomic ensemble (°K) and M is the mass of the isotope (AMU).

When the atom source is heated to a temperature of 1500 °C, the Doppler broadening of the unhindered atoms of the ensemble can be calculated to be 3240 MHz for the 276.3906 nm transition. Since the optical pumping process is intended to pump atoms into the 4d^9^5s ^3^D_2_ (7755.025 cm^−1^) meta-stable state, the Doppler broadening of the transition is not a concern. However, in practice there will be a limit to the Doppler broadening which is governed by the physical dimensions of the self-collimating long canal type atomizer and apertures if any in perpendicular plane to the propagation axis of the effusing atoms.

Doppler broadening of the atomic transition is limited by the angular divergence of the atomic beam. The full angle divergence of the atom source having an aperture diameter “d” and length “l” can be calculated using the expression^19^

29 $$\theta =2{\times {\text{Tan}}}^{-1} \left(\frac{d}{l}\right)$$

In order to account for the Doppler broadening of the atomic transitions, the atom velocities and angular divergence have been segmented into 30 groups each which was sufficient to obtain the convergence of the ionization efficiency values. The segmentation has been done in the following manner. For example, if the full angle divergence is 30°, the angular divergence is varied between the values of − 15° to + 15° with a step size of 1°. Each angular group is further segmented into 30 velocity segments in the range of ± 4 × v_mp_ and the flux of each velocity group is determined by the Eq. (27).

## Results and discussion

**Figure 3:**
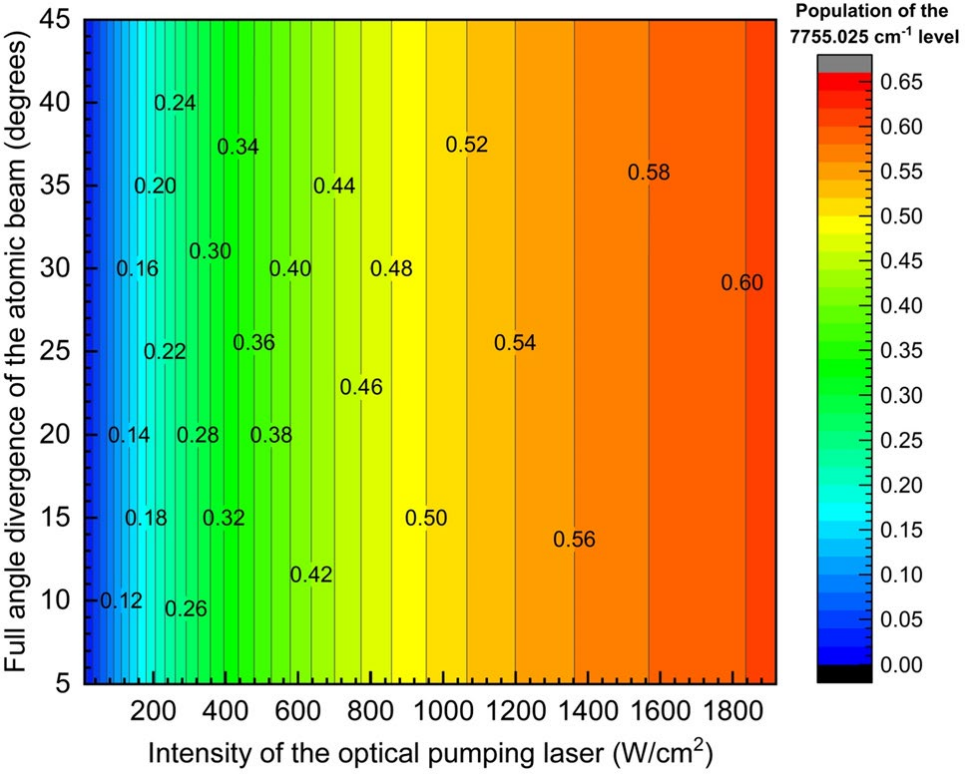
Optical pumping efficiency of ^102^Pd atoms into the 4d^9^5s ^3^D_2_ (7755.025 cm^-1^) meta-stable state. Bandwidth of the optical pumping laser is 10 GHz.

Due to the small isotope shifts of the 276.3906 nm transition (Table 2), it is not possible to obtain any isotopic selectivity in the optical pumping process; therefore, broadband lasers can be utilized. A series of calculations of optical pumping efficiency (i.e. population of the 4d^9^5s ^3^D_2_ (7755.025 cm^−1^) meta-stable state) has been carried out varying the intensity of the optical pumping laser and full angle divergence of the atomic beam and the results are plotted in Fig. 3. The bandwidth of the optical pumping laser was set to 10 GHz for these calculations. From Fig. 3, as expected, optical pumping efficiency increased with an increase in the intensity of the optical pumping laser. It can also be observed that for a given intensity, variation in the full angle divergence of up to a value of 45° did not show any impact on the optical efficiency. This is due to the large bandwidth of the optical pumping laser (10 GHz) which is larger than the Doppler broadening of 2480 MHz at a full angle divergence of the atomic beam of 45°. For a laser intensity of 1800 W/cm^2^, the optical pumping efficiency is found to be 0.60. For a laser with a 30 mm beam diameter and having an intensity of 1800 W/cm^2^ the average power corresponds to 0.38 W (calculated based on the constant intensity laser pulse).

**Figure 4:**
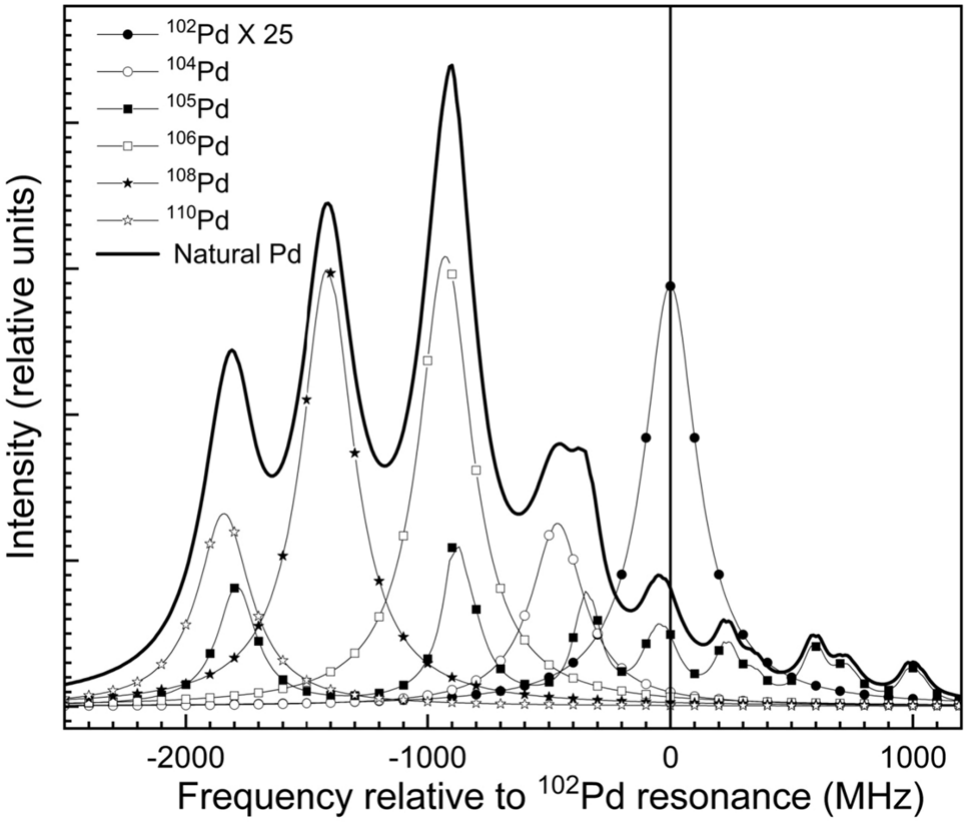
Simulated Doppler free excitation spectrum of 361.0575 nm transition of natural Pd. Intensity of ^102^Pd isotope is multiplied 25 times for better visualization. Please refer to Table 4 for the resonance frequency positions of Pd isotopes. The bandwidth of the excitation laser is taken as 10 MHz and the intensities of both excitation and ionization lasers are 10 W/cm^2^.

The simulated Doppler free frequency spectrum of natural Pd has been computed based on the isotope shifts (Table 2) and the hyperfine structure (Table 3) of ^105^Pd isotopes for the 361.0579 nm transition which is shown in Fig. 4. The resonance frequency positions of the even Pd isotopes and hyperfine transitions of odd ^105^Pd isotope have been tabulated in Table 4. The resonance frequency positions of even isotopes of Pd isotope lie > 450 MHz away from the resonance of the ^102^Pd isotope. Therefore, at low powers, when the bandwidth of the excitation laser is < 200 MHz, the even isotopes are not expected to get ionized significantly. Nevertheless, at high powers, due to the power (saturation) broadening, the even isotopes are also ionized considerably. On the other hand, hyperfine spectrum of the only odd ^105^Pd isotope is spread over 2819 MHz, impeding the selective ionization of ^102^Pd isotope. Particularly the 7/2–7/2 hyperfine transition of ^105^Pd which lies 32 MHz away from the ^102^Pd resonance causes significant overlap. As a result, enrichment of ^105^Pd along with enrichment of ^102^Pd is inevitable. Therefore, the process should be optimized for the depletion of the remaining even isotopes i.e., ^104^Pd, ^106^Pd, ^108^Pd and ^110^Pd.

**Table 3:** Hyperfine structure constants of ^105^Pd isotope for different energy levels of the photoionization scheme.

Energy level	Hyperfine structure constants of ^105^Pd		References
	A (MHz)	B (MHz)	
4d^9^5s ^3^D_2_ (7755.025 cm^−1^)	+ 66.359	− 398.192	^20^
4d^9^5p ^3^P°_1_ (36,180.677 cm^−1^)	− 126.2	6	^11^
4d^9^5p ^3^F°_3_ (35,451.443 cm^−1^)	− 115.3	− 497	^11^

**Table 4:** Table of the resonance frequency positions of Pd isotopes for the 361.0575 nm transition.

Isotope	J1 /F1	J2 /F2	Frequency (MHz)	Intensity	Intensity (normalized to abundance)	Intensity (normalized to ^102^Pd transition)
^110^Pd	2	3	− 1843.0	100.0	0.117	11.47
^105^Pd	4.5	5.5	− 1790.1	28.6	6.37 × 10^–2^	6.25
^108^Pd	2	3	− 1413.6	100.0	0.265	25.98
^106^Pd	2	3	− 927.4	100.0	0.273	26.76
^105^Pd	4.5	4.5	− 882.6	4.4	9.83 × 10^–3^	0.96
^105^Pd	3.5	4.5	− 852.8	19.4	4.33 × 10^–2^	4.24
^104^Pd	2	3	− 465.2	100.0	0.111	10.88
^105^Pd	4.5	3.5	− 363.8	0.4	7.87 × 10^–4^	0.08
^105^Pd	3.5	3.5	− 334.0	6.5	1.44 × 10^–2^	1.41
^105^Pd	3.5	2.5	− 69.6	0.8	1.82 × 10^–3^	0.18
^105^Pd	2.5	3.5	− 32.0	12.2	2.73 × 10^–2^	2.68
^102^Pd	2	3	0.0	100.0	1.02 × 10^–2^	1.00
^105^Pd	2.5	2.5	232.4	6.6	1.47 × 10^–2^	1.45
^105^Pd	2.5	1.5	346.7	1.1	2.55 × 10^–3^	0.25
^105^Pd	1.5	2.5	597.4	6.9	1.53 × 10^–2^	1.50
^105^Pd	1.5	1.5	711.7	5.4	1.21 × 10^–2^	1.18
^105^Pd	1.5	0.5	750.4	1.1	2.36 × 10^–3^	0.23
^105^Pd	0.5	1.5	990.4	3.0	6.61 × 10^–3^	0.65
^105^Pd	0.5	0.5	1029.1	3.7	8.26 × 10^–3^	0.81

Frequency positions of Pd isotopes are referenced to the resonance frequency of the ^102^Pd isotope.

### Effect of power broadening

When isotope shifts of the constituent isotopes and natural abundance of the target are small, isotope selective photoionization is a complex task. The line broadening mechanisms such as Doppler broadening and saturation broadening further adversely affect the process. Therefore, careful control of these parameters is necessary to obtain reasonable isotopic selectivity.

The power (saturation) broadening (Γ_sat_) of an atomic transition can be calculated using the expression

30 $${\Gamma }_{{\text{sat}}}= {\Gamma }_{0 }\sqrt{1+\frac{{{\text{I}}}_{{\text{laser}}}}{{{\text{I}}}_{{\text{sat}}}}}$$

where Γ_0_ is the natural broadening of the transitions; I_laser_ is the intensity of the laser (W/cm^2^); I_sat_ is the saturation intensity of the transition (W/cm^2^).

**Figure 5:**
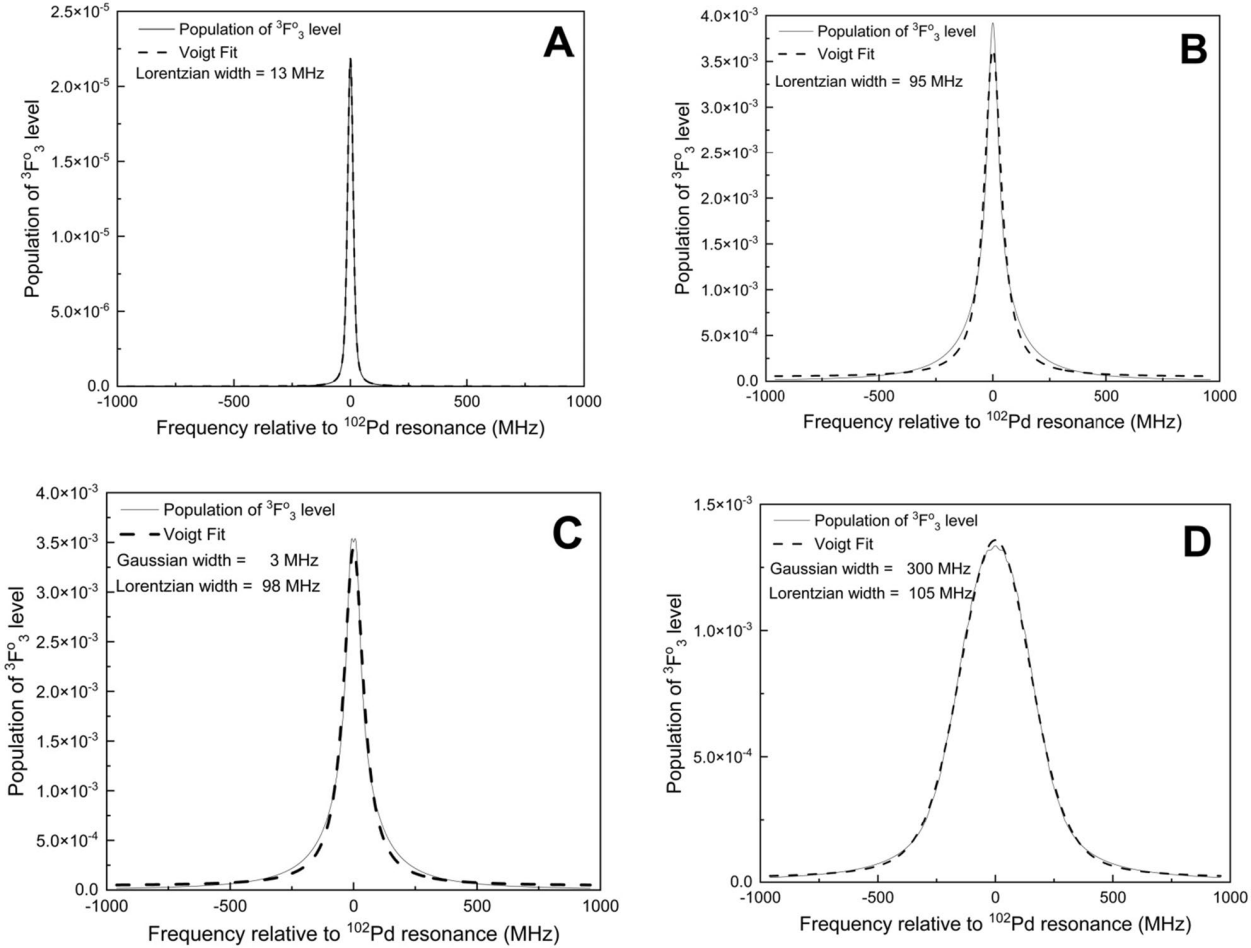
Lineshape of the ^102^Pd isotope for the 361.0575 nm transition. (**A**) Doppler free, bandwidth of the excitation laser is 0 MHz, intensity of the excitation and ionization lasers are 1mW/cm^2^ and 0 W/cm^2^ respectively. (**B**) same as A, except that the intensity of the excitation laser is 10 W/cm^2^. (**C**) same as B, except that the linewidth of the excitation laser is 10 MHz. (**D**) same as C, except that the full angle divergence of the atomic beam is 10°.

The lineshapes calculated using density matrix formalism (Eqs. 4–13 and 16–25) inherently manifest natural broadening and the saturation broadening of the atomic transitions. Line broadening due to laser bandwidth is included through Eq. (14) while the Doppler broadening is included through the flux-velocity distribution of the atomic ensemble. The effect of flux-velocity distribution on the Doppler broadening has been discussed in the earlier section. To illustrate further, we consider the following four cases (Fig. 5A–D).

The lineshape of ^102^Pd has been calculated for the 361.0575 nm transition by setting the bandwidth of the excitation laser to 0 MHz, intensity of the excitation and ionization lasers to 1 mW/cm^2^ and 0 W/cm^2^ respectively under Doppler free condition (i.e. ignoring flux-velocity distribution of atoms). From the transition probability^21^ of 8.2 × 10^7^ s^−1^ for the 361.0575 nm transition, the natural broadening and saturation intensity have been calculated to be 13 MHz and 109 mW/cm^2^ respectively. As the intensity of the excitation laser is low (in comparison to the saturation intensity), the resultant lineshape (Fig. 5A) shows Lorentzian broadening of 13 MHz which corresponds to the natural broadening.

Under the same conditions, when the intensity of the excitation laser is increased to 10 W/cm^2^, the Lorentzian broadening (due to confluence of both natural and saturation broadening) has been found to be 95 MHz (Fig. 5B). The observed value of the saturation broadening (95 MHz) is reasonably close to the value of 126 MHz calculated using Eq. (30). The difference in the saturation broadening values is because of the following reason. Saturation broadening calculated using Eq. (30) is valid for continuous-wave lasers (or flat-top pulsed lasers) while the temporal pulse-shape of the pulsed excitation laser used is Gaussian.

Further, when the bandwidth of the excitation laser is increased from 0 to 10 MHz (Fig. 5C), the resultant line shape showed a Gaussian width of 3 MHz. Finally, when the full angle divergence of the atomic ensemble is set 10° (Fig. 5D), the resultant lineshape shows a Gaussian width of 300 MHz.

When the ionization laser is introduced in the photoionization process; the saturation broadening increases further due to apparent reduction in the lifetime of the excited state. De Groote et al.^22^ have studied the influence of intensity of the ionization laser on the saturation broadening of the atomic transitions. It has been shown by them that temporal delay between the excitation and the ionization lasers results in improved resolution in atomic spectra.

Therefore the intensity of the excitation and ionization lasers needs to be optimally controlled to obtain high ionization efficiency without significant sacrifice in the degree of enrichment.

In order to find optimal values for bandwidth of excitation laser and intensity of the excitation and ionization laser, a series of calculations of the ionization efficiency of constituent isotopes have been carried out varying the intensity of the excitation and ionization lasers under Doppler free conditions (i.e. ignoring the velocity distribution of atoms). From the calculated ionization efficiency values of the constituent isotopes, the degree of enrichment of ^102^Pd has been calculated using the expression

31 $$\mathrm{Degree of enrichment }\left(\mathrm{\%}\right)\mathrm{ of }{}^{102}\mathrm{Pd }=\left\{\frac{{\upeta }_{{102}_{{\text{Pd}}}}{.{\text{A}}}_{{102}_{{\text{Pd}}}}}{\sum_{x}^{all isotopes}{\upeta }_{{{\text{x}}}_{{\text{Pd}}}}{.{\text{A}}}_{{{\text{x}}}_{{\text{Pd}}}}}\right\}*100$$

where A = Initial fractional abundance of the isotope and η = Ionization efficiency.

**Figure 6:**
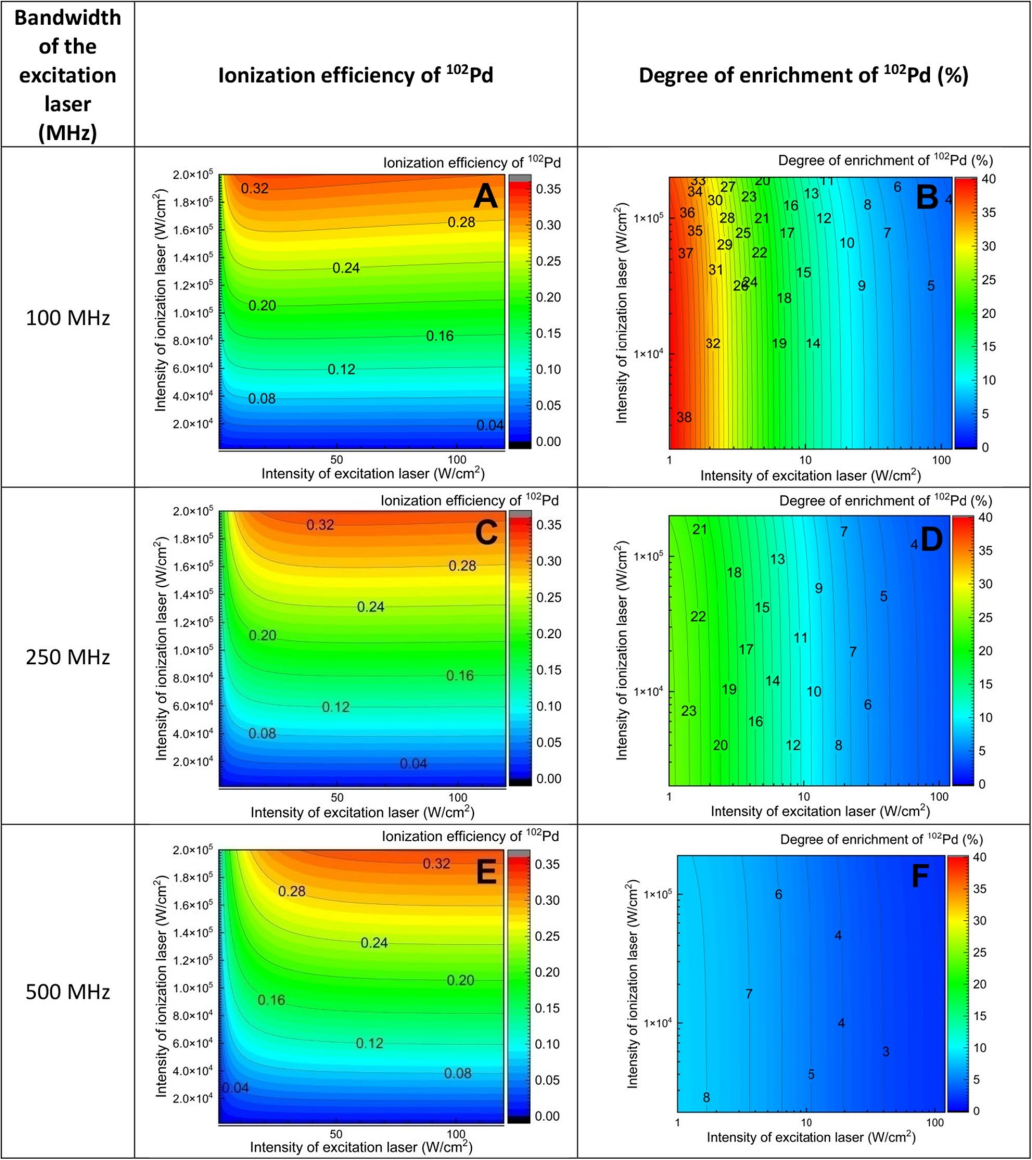
Dependence of ionization efficiency and degree of enrichment of ^102^Pd isotope on the intensity of the excitation and ionization lasers under Doppler free conditions.

The obtained ionization efficiency and the degree of enrichment of ^102^Pd have been plotted in Fig. 6 for different bandwidths. From Fig. 6, the following conclusions can be made.

1. The ionization efficiency of ^102^Pd isotope is largely invariant with increase in the intensity of the excitation laser (Fig. 6A, C, E). This is due to the low saturation power of 109 mW/cm^2^ for the strong $4{d}^{9}5s {}^{3}{D}_{2} \stackrel{361.0575 nm}{\to }4{d}^{9}5p {}^{3}{F}_{3}^{o}$ transition.
2. As expected for any non-resonant ionization process, the ionization efficiency of ^102^Pd isotope increases with the increase in the intensity of the ionization laser (Fig. 6A, C, E).
3. The degree of enrichment falls gradually with the increase in the intensity of the excitation laser while it is invariant to the intensity of the ionization laser (Fig. 6B, D, F) which is due to the large power broadening induced by the excitation laser.
4. When the bandwidth of the excitation laser is 500 MHz, the degree of enrichment falls to < 8%. On the whole, a bandwidth of 100 MHz seems to be an optimum value for obtaining a meaningful enrichment of ^102^Pd.

### Effect of Doppler broadening and laser bandwidth

As mentioned earlier, calculations of ionization efficiency and degree of enrichment described in Fig. 6 have been carried out ignoring the Doppler broadening of the atomic system. However, in real situations, the atomic lines are Doppler broadened due to finite divergence angle of the atoms and velocity distribution. When the atom source is heated to a temperature of 1500 °C, the Doppler broadening of the unhindered atoms of the ensemble will be 2480 MHz for the 361.0575 nm transition. Since the Doppler broadening is much larger than the frequency difference between the resonances of the target and non-target isotopes, the Doppler broadening along the laser propagation axis must be reduced.

The Doppler broadening along the laser axis can be curtailed by incorporating additional collimators along the atomic beam propagation axis which determines the full angle divergence of the atomic beam. In this case, the flux of the atoms having higher divergence is inhibited to enter the laser-atom interaction region. However, this results in loss of throughput of the system. Alternatively, Doppler broadening of the atomic transition can also be controlled using long canal type atomizers having full angular divergence as per Eq. (29). In order to account for the Doppler broadening, as discussed previously, both full angle divergence and the velocity distribution are segmented into 30 groups each for the calculations.

**Figure 7:**
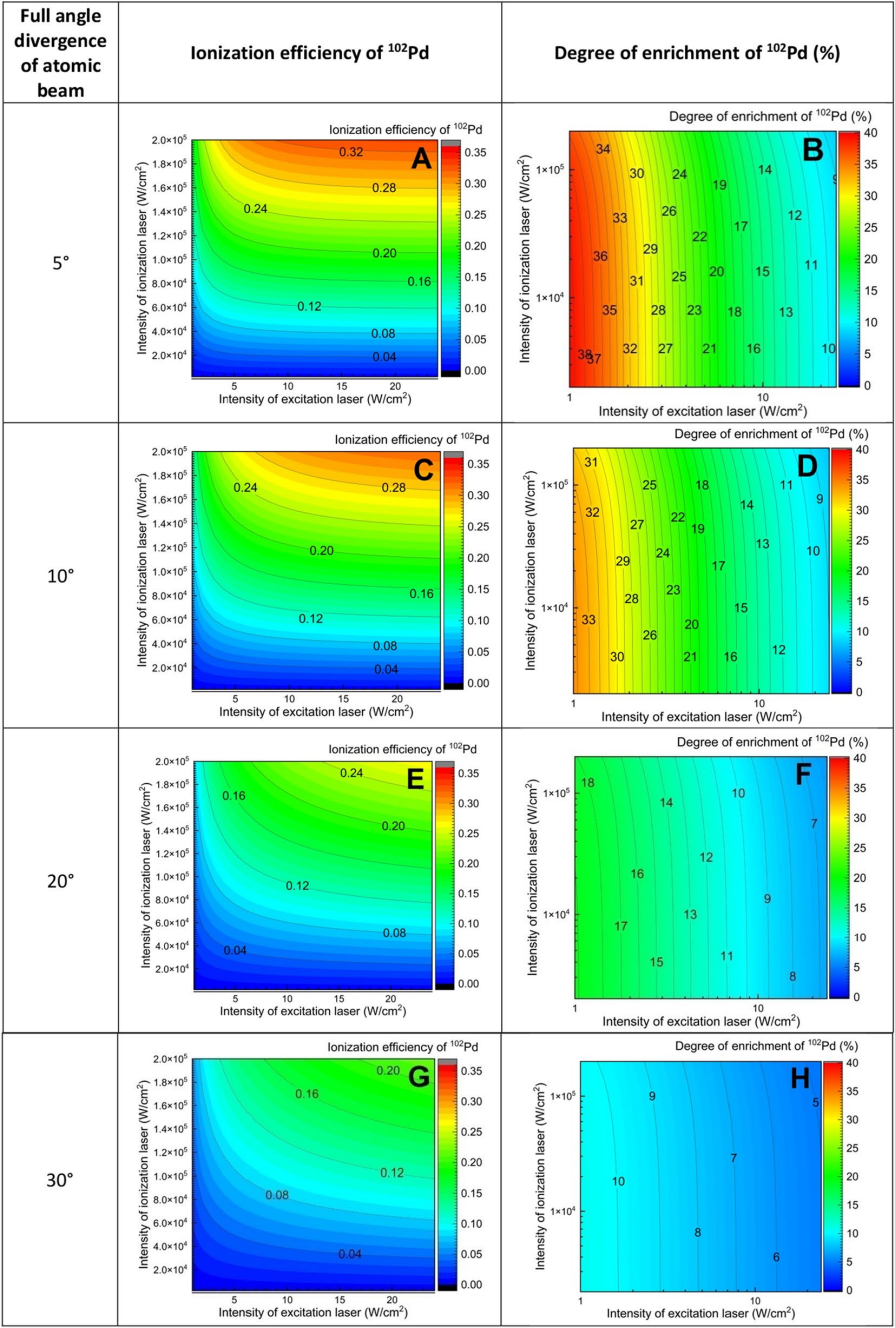
Plot of the dependence of ionization efficiency and degree of enrichment of ^102^Pd isotope on the intensity of the excitation, ionization lasers and the full angle divergence of the atoms. Bandwidth of the excitation laser is 100 MHz.

The overall lineshape of a resonance is a result of complex confluence of the bandwidth of the excitation laser, Doppler broadening and the power broadening which dictate both ionization efficiency and the degree of enrichment. A series of calculations of ionization efficiency and degree of enrichment of ^102^Pd have been carried out varying the intensity of the excitation and ionization lasers as well as full angle divergence of atoms. The results are shown in Fig. 7. The dependence of both the ionization efficiency and degree of enrichment of ^102^Pd on laser intensities showed similar pattern like in Doppler free conditions (Fig. 7 A and B) except that in the present case, both ionization efficiency and degree of enrichment dropped further which can be attributed to the Doppler broadening. With increase in the full divergence of atoms to ≥ 20°, the degree of enrichment dropped drastically. Therefore, it is required to limit the full angle divergence of the atoms to 10° to obtain adequate enrichment in the laser isotope separation process.

### Effect of charge exchange collisions

Charge exchange collisions have an adverse effect on the enrichment process. Photoions generated during laser excitation process undergo charge exchange collisions with neutral atoms prior to the collection at the ion collector.

Resonant charge exchange cross-section can be calculated using the following formula^23^

32 $$\sigma \left(v\right)=\left(1.81\times {10}^{-14}-2.12\times {10}^{-15}{.{\text{log}}}_{10}v\right).{\left(\frac{IP}{13.6}\right)}^{-1.5}$$

where v is the velocity of the ion in cm/s and IP is the ionization potential of the element in eV.

For the most probable atomic velocity of 537.507 m/s for Pd at 1500 °C, the resonant charge exchange cross-section has been calculated to be 1.6 × 10^–14^ cm^2^ which is in good agreement with the value reported by Smirnov^24^.

The charge exchange probability can be calculated using the expression

33 $$Charge exchange probability p=1-{e}^{-\sigma dN}$$

Where σ is the resonant charge exchange cross-section (cm^2^), d is the distance traversed by the photoions prior to the collection at the collector (cm) and N is the number density of the atoms (atoms /cm^3^). For d = 3 cm, N = 1 × 10^12^ atoms/cm^3^ (0.24 μbar); the charge exchange probability is 4.7%.

**Figure 8:**
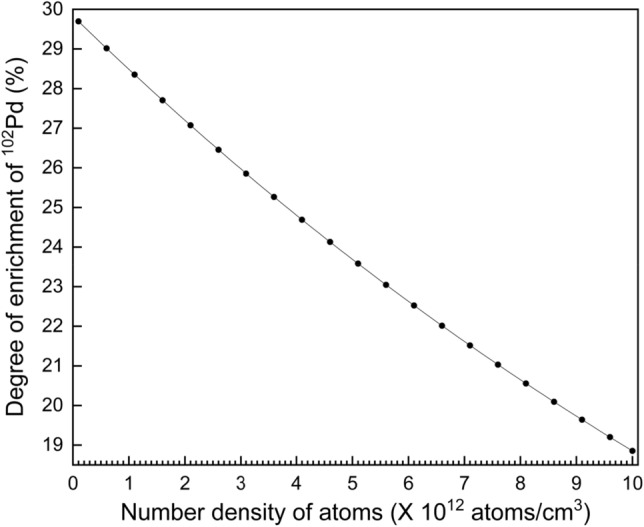
Plot of the dependence of the degree of enrichment on the number density of atoms in the laser-atom interaction region. Laser bandwidth is 100 MHz, full angle divergence of atoms is 10° and the intensity of the excitation and ionization lasers are 1.5 W/cm^2^ and 1 × 10^5^ W/cm^2^ respectively.

Computations of the degree of enrichment have been carried out varying the number density of atoms in the laser-atom interaction region and the results are plotted in Fig. 8. A gradual reduction in the degree of enrichment with increase in the number density has been observed. At a number density of 5 × 10^12^ atoms/cm^3^ (1.22 μbar), the degree of enrichment was found to be about 23.7%. Of course it is also possible to choose any other number density based on the requirement of the degree of enrichment.

### Production rate

Production rates can be calculated using the following equation

34 $$P \left(\frac{{\text{g}}}{{\text{hour}}}\right)=2.827\times {10}^{3}\times \left({b}^{2}.p.l.d.A.f.{\eta }_{o}.{\eta }_{i}.i.n.\frac{M}{{N}_{A}}.PRF\right)$$

where b is the laser beam diameter (cm), *p* is the fractional population of the ground level, l is the length of the laser-atom interaction region (cm), d is the number density of atoms in the interaction region (atoms/cm^3^), A is the fractional abundance of the target isotope, f is the fractional flux (flux relative to the flux of unhindered atomic beam), η_o_ is the optical pumping efficiency, η_i_ is the ionization efficiency (both derived from the density matrix calculations), *i* is the irradiation probability, n is the number of passes of the laser beam through the laser-atom interaction region, M is the atomic mass of the target isotope (AMU), N_A_ is the Avogadro number (6.02214076 × 10^23^) and PRF is the pulse repetition frequency of the lasers (Hz).

For the values of b = 3 cm, p = 1, l = 90 cm, d = 5 × 10^12^ atoms/cm^3^ (1.22 μbar), A = 0.0102, f = 1, η_o_ = 0.6, η_i_ = 0.094, i = 1, n = 1, M = 102, PRF = 1 kHz, the production rate has been calculated to be 1.1 mg/h (or 27 mg/day). A summary of the optimum system parameters for the separation of ^102^Pd isotope is shown in Table 5.

**Table 5:** A brief summary of the optimized parameters for the laser isotope separation of ^102^Pd.

Parameter	Value
Common parameters of laser system	
Pulse width of the laser(s)	30 ns
Pulse repetition frequency	1 kHz
Laser beam diameter	30 mm
Optical pumping process	
Wavelength	276.3906 nm
Bandwidth	Broadband (~ 10 GHz)
Intensity	1800 W/cm^2^ (average power 0.38 W)
Photoionization process	
Excitation laser wavelength	361.0575 nm
Bandwidth	100 MHz
Intensity	1.5 W/cm^2^ (average power 0.32 mW)
Ionization laser wavelength	< 341 nm
Intensity	1 × 10^5^ W/cm^2^ (21.2 W)
Source parameters	
Evaporation temperature	1500°
Full angle divergence of the atomic beam	10°
Number density in the laser-atom interaction region	5 × 10^12^ atoms/cm^3^ (1.22 μbar)
Results	
Optical pumping efficiency	0.60
Photoionization efficiency	0.094
Overall efficiency	5.6 × 10^–2^
Production rate	1.1 mg/h (27 mg/day)

### Irradiation of enriched Pd

When the laser isotope separation of Pd is carried out under the conditions described in Table 5, the abundances of the enriched isotopic mixture is ^102^Pd (23.69%), ^104^Pd (13.98%), ^105^Pd (40.49%), ^106^Pd (11.08%), ^108^Pd (7.74%) and ^110^Pd (3.03%). The utility of irradiated enriched Pd for nuclear medicine is evaluated as discussed below.

When the Pd isotope mixture is irradiated in a nuclear reactor, they undergo (n, γ) reactions and produce daughter nuclides. The radioactivity production equations^25^ for Pd isotopes can be written as given below.

35 $${\int }_{0}^{t}d{N}_{102}= -{\int }_{0}^{t}{N}_{102}.{\sigma }_{102}.\varphi .dt$$

36 $${\int }_{0}^{t}d{N}_{103}={\int }_{0}^{t}{N}_{102}.{\sigma }_{102}.\varphi .dt-{\int }_{0}^{t}{N}_{103}.{\sigma }_{103}.\varphi .dt- \frac{ln2}{{\left[{t}_{1/2}\right]}_{103}}{\int }_{0}^{t}{N}_{103}.dt$$

37 $${\int }_{0}^{t}d{N}_{104}={\int }_{0}^{t}{N}_{103}.{\sigma }_{103}.\varphi .dt-{\int }_{0}^{t}{N}_{104}.{\sigma }_{104}.\varphi .dt$$

38 $${\int }_{0}^{t}d{N}_{105}={\int }_{0}^{t}{N}_{104}.{\sigma }_{104}.\varphi .dt-{\int }_{0}^{t}{N}_{105}.{\sigma }_{105}.\varphi .dt$$

39 $${\int }_{0}^{t}d{N}_{106}={\int }_{0}^{t}{N}_{105}.{\sigma }_{105}.\varphi .dt-{\int }_{0}^{t}{N}_{106}.{\sigma }_{106}.\varphi .dt$$

40 $${\int }_{0}^{t}d{N}_{107}={\int }_{0}^{t}{N}_{106}.{\sigma }_{106}.\varphi .dt-{\int }_{0}^{t}{N}_{107}.{\sigma }_{107}.\varphi .dt- \frac{ln2}{{\left[{t}_{1/2}\right]}_{107}}{\int }_{0}^{t}{N}_{107}.dt$$

41 $${\int }_{0}^{t}d{N}_{108}={\int }_{0}^{t}{N}_{107}.{\sigma }_{107}.\varphi .dt-{\int }_{0}^{t}{N}_{108}.{\sigma }_{108}.\varphi .dt$$

42 $${\int }_{0}^{t}d{N}_{109}={\int }_{0}^{t}{N}_{108}.{\sigma }_{108}.\varphi .dt-{\int }_{0}^{t}{N}_{109}.{\sigma }_{109}.\varphi .dt- \frac{ln2}{{\left[{t}_{1/2}\right]}_{109}}{\int }_{0}^{t}{N}_{109}.dt$$

43 $${\int }_{0}^{t}d{N}_{110}={\int }_{0}^{t}{N}_{109}.{\sigma }_{109}.\varphi .dt-{\int }_{0}^{t}{N}_{110}.{\sigma }_{110}.\varphi .dt$$

where N_i_ is the amount of Pd isotope, σ_i_ is the thermal neutron absorption cross-section of the isotope (cm^2^), φ is the thermal neutron flux of the reactor (neutrons/cm^2^/sec) and t is the irradiation time.

**Figure 9:**
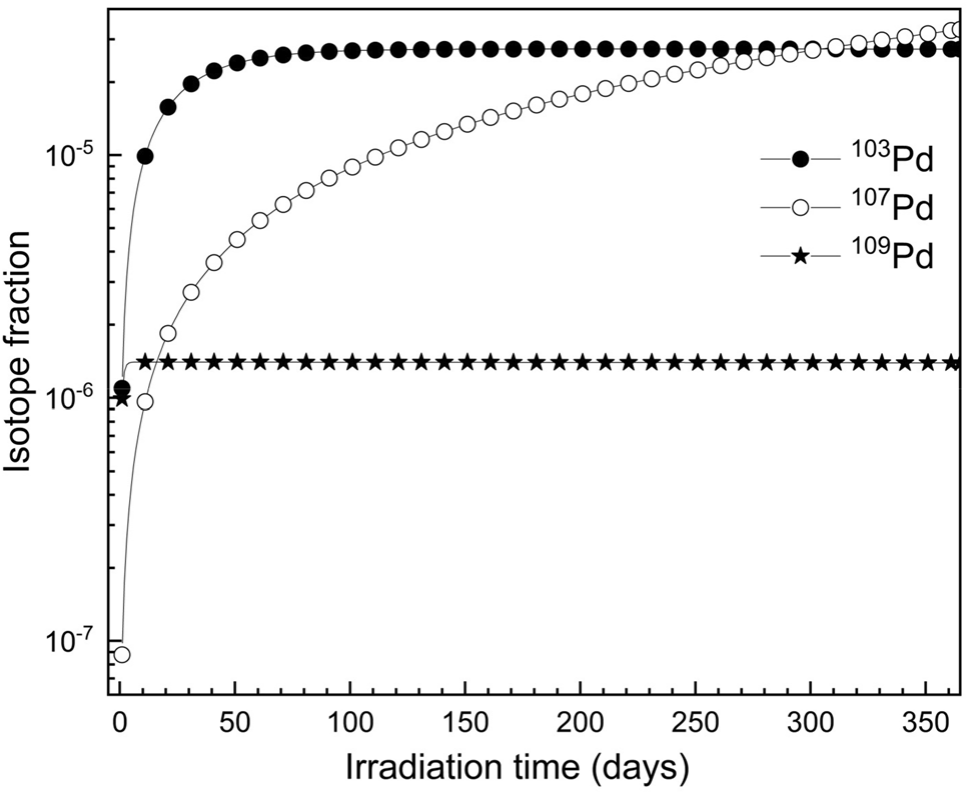
Isotope fractions of radioactive isotopes of Pd with irradiation time. Thermal neutron flux of the reactor is taken as 3 × 10^13^ neutrons/cm^2^/s and the cooling time is 0 h.

**Figure 10:**
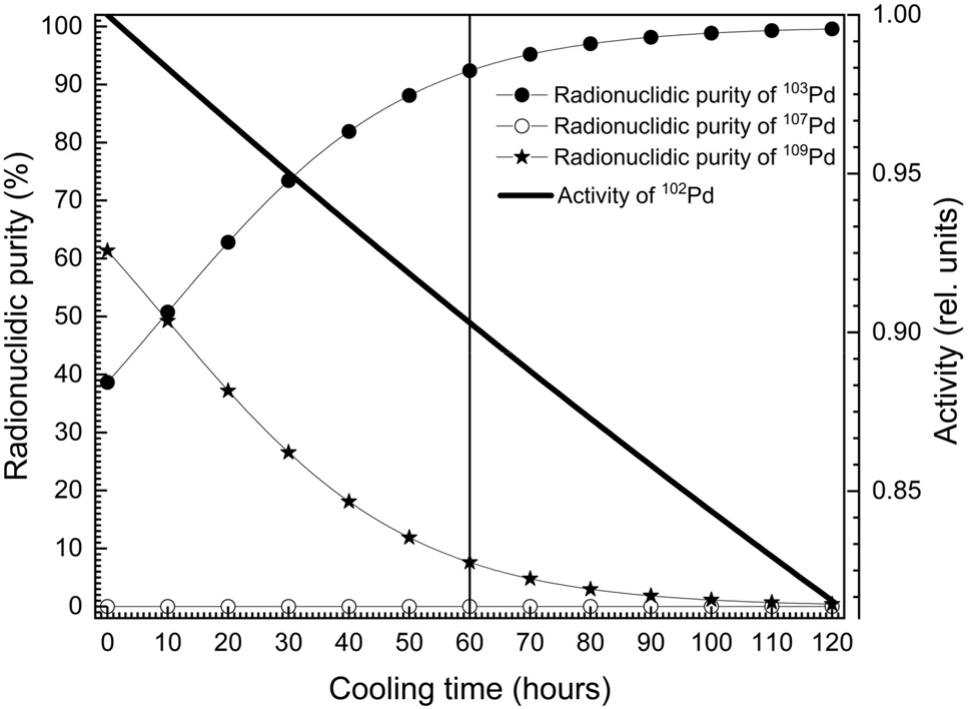
Plot of the variation in radionuclidic purity of ^103^Pd, ^107^Pd and ^109^Pd isotopes with cooling time. The irradiation time of the enriched isotopic mixture is 60 days in a low-flux (3 × 10^13^ neutrons/cm^2^-sec) nuclear reactor. Decay of the activity of ^102^Pd as a function of the cooling time is also plotted.

When the enriched isotopic mixture is irradiated in a low-flux nuclear reactor (3 × 10^13^ neutrons/cm^2^-sec), radioactive ^107^Pd and ^109^Pd isotopes are also produced along with the desired ^103^Pd isotope (Fig. 9). It can be observed from Fig. 9 that, it requires about 60 days of irradiation in order to obtain maximum production yield of ^102^Pd isotope. At the end of irradiation (60 days), the amount of radioactive isotopes produced is 25 μg of ^102^Pd, 5.3 μg of ^107^Pd and 1.4 μg of ^109^Pd. Since the radioactive isotopes decay at different rates due to the vast difference in their half-lives, their relative fractions also vary with time; therefore, the radionuclidic purity of the isotopes varies with time. Radionuclidic purity of radioactive isotopes of Pd has been has been calculated varying the cooling time using the Eqs. (1 and 2) and the results are plotted in Fig. 10.

At the end of irradiation time, the radionuclidic purity of ^103^Pd, ^107^Pd and ^109^Pd are 38.6%, 5.6 × 10^–6^% and 61.4% respectively. At the outset the radionuclidic purity seems to be low. However, it should be noted that the ^109^Pd (T_1/2_ = 13.59 h) isotope dies down quickly due to its relatively low half-life as compared to ^103^Pd. As a result the radionuclidic purity of ^103^Pd increases with cooling time (Fig. 10). After a cooling period of 60 h, the radionuclidic purity of ^102^Pd increases to > 92%; while the loss in activity during this period is just 10%. Therefore, enrichment of ^102^Pd using atomic vapor laser isotope separation process described above can be used for the production of ^103^Pd isotope for cancer therapy.

## Conclusions

The feasibility of laser isotope separation of ^102^Pd through pulsed laser optical pumping followed by isotope selective photoionization has been studied through density matrix formalism. The effect of various parameters such as bandwidth of the excitation lasers, intensity of the lasers and Doppler broadening of the atomic ensemble on the efficiency of optical pumping and isotope selective photoionization have been evaluated. The optimum number density in the laser-atom interaction has been derived from the studies of the effect of charge exchange collisions on the degree of enrichment. It has been shown that it is possible to enrich the ^102^Pd isotope up to ~ 23.7% at a production rate of 1.1 mg /h. The achievable degree of enrichment through this photoionization scheme is higher than previously reported laser isotope separation^12,13^. The radionuclidic purity analysis of irradiated enriched mixture has been found to be suitable for medical applications.

The overall efficiency of the laser isotope separation process has been found to be 5.6 × 10^–2^. Though the optical pumping efficiency (0.6) is adequately high, the low efficiency (9.4 × 10^–2^) of the isotope selective photoionization process remains a primary impediment at present. This also highlights the need for more experimental work on the suitable second step excitation transitions originating from the 4d^9^5p ^3^F°_3_ (35,451.443 cm^−1^) and autoionization transitions from the connected levels which makes the laser isotope separation more efficient.

## Data Availability

The datasets used and/or analysed during the current study available from the corresponding author on reasonable request.

## References

[1] Bush, R. P. Recovery of platinum group metals from high level radioactive waste. *Platin. Metals Rev.* **35**, 202–208 (1991). 10.1595/003214091X354202208

[2] Pokhitonov, Yu. A. & Tananaev, I. G. Prospects for the use of Palladium from NPP spent nuclear fuel and ways to design the technology of its recovery at a radiochemical enterprise. *Radiochemistry* **64**, 270–279 (2022). 10.1134/S106636222203002X

[3] Chen H. L. Laser cleanup of Pt group metals, Report: UCID-18837 TRN: 81-001926, Lawrence Livermore National Lab (1980).

[4] Geldhof, S. *et al.* Impact of nuclear deformation and pairing on the charge radii of palladium isotopes. *Phys. Rev. Lett.* **128**, 152501. 10.1103/PhysRevLett.128.152501 (2022). 35499902 10.1103/PhysRevLett.128.152501

[5] Evaluated Nuclear Structure Data Files (ENSDF), IAEA. (Weblink: https://www-nds.iaea.org/relnsd/vcharthtml/VChartHTML.html).

[6] Stone, N. J. Table of nuclear magnetic dipole and electric quadrupole moments. *At. Data Nucl. Data Tables* **90**, 75–176 (2005). 10.1016/j.adt.2005.04.001

[7] Blasko, J. C. *et al.* Palladium-103 brachytherapy for prostate carcinoma. *Int. J. Radiat. Oncol. Biol. Phys.* **46**, 839–850 (2000). 10705004 10.1016/S0360-3016(99)00499-X

[8] Reichstein, D. A. & Brock, A. L. Radiation therapy for uveal melanoma: a review of treatment methods. *Curr. Opin. Ophthalmol.* **32**, 183–190 (2021). 33770014 10.1097/ICU.0000000000000761

[9] Duncan, C. L. & Krane, K. S. Neutron capture cross section of ^102^Pd. *Phys. Rev.* **71C**, 054322 (2005).

[10] Van Duijn, E. J. & Witte, S. Hyperfine structure and isotope shift measurements on 4d^10^^1^S_0_ → 4d^9^ 5p J=1 transitions in Pd I using deep-UV cw laser spectroscopy. *Eur. Phys. J. D At. Mol. Opt. Plasma Phys.* **19**, 25–29 (2002).

[11] Sarina Geldhof developments for high-resolution laser spectroscopy and application to palladium isotopes. Doctoral Thesis, University of Jyväskylä (2020).

[12] Derzhiev, V. I. *et al.* Two-step photoionisation of palladium. *Quantum Electron.* **32**, 619–622 (2002). 10.1070/QE2002v032n07ABEH002257

[13] Derzhiev, V. I. *et al.* Isotope—selective photoionisation of palladium. *Quantum Electron.* **33**, 553–558 (2003). 10.1070/QE2003v033n06ABEH002456

[14] Tkachev, A. N. & Yakovlenko, S. I. On laser rare-isotope separation. *Quantum Electron.* **33**, 581–592 (2003). 10.1070/QE2003v033n07ABEH002465

[15] Geldhof, S. *et al.* Collinear laser spectroscopy of stable palladium isotopes at the IGISOL facility. *Hyperfine Interact.* **241**, 41 (2020). 10.1007/s10751-020-01713-3

[16] Shore, B. W. *The Theory of Coherent Atomic Excitation: Simple Atoms and Fields* Vol. 1 (Wiley, 1990). 10.1126/science.250.4987.1603 17818288

[17] Haynes, W. M. (ed.) *CRC Handbook of Chemistry and Physics* 97th edn. (Taylor & Francis Group, 2016–2017).

[18] Xu, H. L. *et al.* Radiative lifetimes, branching fractions and oscillator strengths in Pd I and the other solar palladium abundance. *Astron. Astrophys.* **452**, 357–362. 10.1051/0004-6361:20054610 (2006). 10.1051/0004-6361:20054610

[19] Clausing, P. Über die Strahlformung bei der Molekularströmung. *Z. Phys.* **66**, 471–476. 10.1007/BF01402029 (1930). 10.1007/BF01402029

[20] Channappa, K. H. & Pendlebury. J. M. Hyperfine structure measurements in some low-lying multiplets of 47Ti, 49Ti, 59Co and 105Pd. In *Proceedings of the Physical Society* Vol. 86 1145 (1965).

[21] Kurucz. R. L. Atomic spectral line database from CD-ROM 23 available at https://www.cfa.harvard.edu/amp/ampdata/kurucz23/sekur.html (1995).

[22] de Groote, R. P. *et al.* Efficient, high-resolution resonance laser ionization spectroscopy using weak transitions to long-lived excited states. *Phys. Rev.* **95A**, 032502 (2017). 10.1103/PhysRevA.95.032502

[23] Sakabe, S. & Izawa, Y. Simple formula for the cross sections of resonant charge transfer between atoms and their positive ions at low impact velocity. *Phys. Rev.* **45A**, 2086–2088 (1992). 10.1103/PhysRevA.45.2086 9907203

[24] Smirnov, B. M. Tables for cross sections of the resonant charge exchange process. *Phys. Scr.* **61**, 595–602 (2000). 10.1238/Physica.Regular.061a00595

[25] Friedlander, G., Kennedy, J. W., Macias, E. S. & Miller, J. M. *Nuclear and Radiochemistry* 3rd edn. (John Wiley & Sons, 1981).

